# Metabolic Engineering of *Saccharomyces cerevisiae* for Efficient Retinol Synthesis

**DOI:** 10.3390/jof9050512

**Published:** 2023-04-26

**Authors:** Xuan Wang, Xianhao Xu, Jiaheng Liu, Yanfeng Liu, Jianghua Li, Guocheng Du, Xueqin Lv, Long Liu

**Affiliations:** 1 Key Laboratory of Carbohydrate Chemistry and Biotechnology, Ministry of Education, Jiangnan University, Wuxi 214122, China; 2 Science Center for Future Foods, Jiangnan University, Wuxi 214122, China; 3 Food Laboratory of Zhongyuan, Jiangnan University, Wuxi 214122, China

**Keywords:** vitamin A, retinol, *Saccharomyces cerevisiae*, retinol dehydrogenase, olive oil

## Abstract

Retinol, the main active form of vitamin A, plays a role in maintaining vision, immune function, growth, and development. It also inhibits tumor growth and alleviates anemia. Here, we developed a *Saccharomyces cerevisiae* strain capable of high retinol production. Firstly, the de novo synthesis pathway of retinol was constructed in *S. cerevisiae* to realize the production of retinol. Second, through modular optimization of the metabolic network of retinol, the retinol titer was increased from 3.6 to 153.6 mg/L. Then, we used transporter engineering to regulate and promote the accumulation of the intracellular precursor retinal to improve retinol production. Subsequently, we screened and semi-rationally designed the key enzyme retinol dehydrogenase to further increase the retinol titer to 387.4 mg/L. Lastly, we performed two-phase extraction fermentation using olive oil to obtain a final shaking flask retinol titer of 1.2 g/L, the highest titer reported at the shake flask level. This study laid the foundation for the industrial production of retinol.

## 1. Introduction

Vitamin A (retinoids) is a fat-soluble vitamin essential for normal metabolism in humans; it is also known as a “beauty vitamin” and “anti-dry eye vitamin” [1,2,3]. Retinol, the main active form of vitamin A, promotes growth and development and collagen regeneration, maintains the normal shape and function of bones and epithelial tissues, and improves vision and immunity. It is widely used in foods, medicines, and animal feed additives [4]. At present, retinol is mainly synthesized through natural extraction and chemical synthesis [5]. However, retinol extraction from animal sources is decentralized, complicated, and costly [6]. The chemical synthesis method [7] consumes high energy and has a high technical threshold. Microbial cell factories have recently been constructed to effectively synthesize several valuable chemicals [8]. Microbial synthesis has many advantages, including short cycles, low cost, mild fermentation conditions, high safety, strong controllability, and climate independency.

In recent years, significant progress has occurred in vitamin A biosynthesis. Sun et al. used xylose as a carbon source in *Saccharomyces cerevisiae* to reduce ethanol production as a by-product, thereby promoting retinol accumulation. They obtained 55.31 mg/L of the precursor retinal and retinol with dodecane two-phase extraction using shake-flask fermentation [9]. The physiological activity of retinol is higher than that of retinal. To maximize the conversion of retinal into retinol, Lee et al. introduced the human *RDH12* gene into *S. cerevisiae*, and the resultant strain selectively produced retinol at a titer of 123.1 mg/L via shake-flask fermentation [10]. However, retinol is easily oxidized during fermentation. Hyemin found that oxidative damage was mainly responsible for retinol degradation inside the cells, which was more efficiently mitigated using a combination of Tween-80 and the antioxidant BHT as an extraction agent than dodecane [11]. The retinol titer reached 437 mg/L in the shake-flask fermentation. Hu et al. achieved a retinol titer of 443.43 mg/L at the shake flask level in *S. cerevisiae* by increasing the supply of NADPH, screening and overexpressing endogenous retinol dehydrogenase, and conducting two-phase fermentation with the addition of antioxidant [12]. Although these methods increased the retinol titer in *S. cerevisiae*, there are still some problems that limit its production; the metabolic pathway is complex and has not been systematically optimized, retinal is transported out of the cell, which hinders the synthesis of retinol, and the conversion efficiency of retinol from retinal is low.

With the development of synthetic biology, several methods have been developed to solve metabolic regulation problems. Modular metabolic engineering can be effectively applied to systematically optimize complex metabolic networks. For example, Chen et al. divided the fumaric acid synthesis pathway into reduction, oxidation, and byproduct modules. They optimized each module step-by-step at the transcription and translation levels using multiple regulatory means, which increased the final fumaric acid titer by 487% to 33.1 g/L [13]. Transporter engineering can be effectively applied for product export or intermediate metabolite accumulation. Hexose transporter engineering in *S. cerevisiae* is an effective strategy for constructing glucose-insensitive xylose transporters to efficiently produce lignocellulosic bioethanol [14]. Semi-rational transformation of enzymes is an effective enzyme engineering transformation approach that improves the catalytic efficiency of key enzymes. The enzyme activity of gamma-humulene synthetase was enhanced by subjecting 19 amino acids in its active center to saturation mutation and fragment recombination [15]. Therefore, the synthetic biology methods mentioned above provide effective strategies for efficient retinol biosynthesis.

In this study, we used inducible promoters to heterologously express genes related to retinol synthesis in *S. cerevisiae* to obtain retinol-producing recombinant strains. The retinol metabolism pathway was divided into five modules for system metabolic regulation through modular metabolic engineering. Next, the transporter proteins involved in the export of retinal were knocked out to reduce its outflow. Then, to improve the conversion rate of retinal to retinol, we mined and overexpressed the endogenous *S. cerevisiae* retinol dehydrogenase, and the activity of the oxidoreductase ENV9 was enhanced using a selective semi-rational design. Lastly, food-grade olive oil was used to replace dodecane for retinol extraction, and 1.21 g/L retinol was obtained using shake-flask fermentation, indicating high-efficiency retinol production.

## 2. Materials and Methods

### 2.1. Culture Medium and Reagents

*Escherichia coli* strains were grown overnight in Luria–Bertani (LB) medium (5 g/L yeast powder, 10 g/L peptone, 10 g/L sodium chloride, and 0.1 mg/mL ampicillin; 20 g/L agar powder was added to the solid medium). *S. cerevisiae* strains were cultivated in Yeast Peptone Dextrose (YPD) medium (10 g/L yeast powder, 20 g/L peptone, and 20 g/L glucose; 20 g/L agar powder was added to the solid medium) to obtain the seed liquid, which was transferred into the fermentation medium (50 g/L soybean peptone, 25 g/L anhydrous glucose, 25 g/L sucrose, 25 g/L glycerol, 0.6 g/L dipotassium hydrogen phosphate, and dodecane) [16]. To select the *S. cerevisiae* strains containing screening markers, synthetic dropout (SD-Trp/His/Leu) solid medium (20 g/L glucose, 50 mg/L histidine/tryptophan/tryptophan, 50 mg/L leucine/leucine/histidine, 50 mg/L uracil, 20 g/L agar, and 6.7 g/L yeast nitrogen source without amino acid) was used.

The media were purchased from Sangon (Shanghai, China), and PrimeSTAR HS DNA polymerase was purchased from Takara (Otsu, Japan). DNA gel purification and plasmid extraction kits were purchased from Thermo Scientific (Wilmington, NC, USA). Heterologous genes were synthesized by GENEWIZ (Suzhou, China). 5-Fluoroorotic acid (5-FOA), amino acids, and squalene, β-carotene, retinal, and retinol standards were purchased from Solarbio (Beijing, China). The antioxidants butylated hydroxytoluene (BHT), propyl gallate (PG), ethoxy quinoline (EQ), tert-butylhydroquinone (TBHQ), olive oil, and dodecane were purchased from Aladdin (Shanghai, China). DNA sequencing was performed by GENEWIZ (Suzhou, China).

### 2.2. Construction of Plasmids and Strains

Four initial plasmids (pY13/14/15, pY26-Cre) were prepared to construct the gene expression box. The promoter and terminator were amplified from the genome of *S. cerevisiae* CEN PK2-1C. All plasmids were constructed in *E. coli* JM109. The target fragments and supporter were obtained via polymerase chain reaction (PCR), transformed into competent *E. coli*, plated on solid LB medium with 100 µg/mL ampicillin, and cultured at 37 °C for 12 h. After selecting the transformed colonies, the plasmids were extracted for sequencing verification. Supplementary Table S1 lists the plasmids used in this study.

Previous studies obtained the strain S6 based on *S. cerevisiae* CEN.PK2-1C to enhance the mevalonate synthesis pathway. In this study, the *S. cerevisiae* S6 strain was used as the chassis strain. As *S. cerevisiae* has a strong homologous recombination ability, the Cre/loxP system was used for gene knockout and integration. According to the expression preference of *S. cerevisiae*, the heterologous genes *crtE*, *crtB*, *crtI, crtYB*, and *blh* [10] were synthesized and codon-optimized by GENEWIZ. The target heterologous gene fragments, screening tags, and upstream and downstream homologous arms were obtained through PCR and then transformed into *S. cerevisiae* competent S6. *S. cerevisiae* was cultured at 30 °C on an SD agar plate to select the transformants with the nutrient deficiency marker and obtain the retinol-producing strain A01.

The strains involved in this paper are shown in Table 1.

### 2.3. Insertion of Degradation Label

The squalene synthesis pathway is the competitive branch of the precursor farnesyl pyrophosphate (FPP). The fusion of the PEST fragment of the key domain in the cyclin CLN2 at the carboxyl end of the squalene synthetase (ERG9) affects protein stability and reduces ERG9 expression. Therefore, we fused the carboxyl end of ERG9 with CLN2 using the linker GGAAGCGCGGTGGGGGCGAGC and screened the strains that successfully integrated the amino-acid tag for product detection.

### 2.4. Structural Simulation and Molecular Docking

The 3D model of retinol dehydrogenase was obtained from the AlphaFold Protein Structure Database (https://alphafold.ebi.ac.uk/ (accessed on 5 October 2022). The molecular ligand retinal was obtained from PubChem (https://pubchem.ncbi.nlm.nih.gov/ (accessed on 5 October 2022) and converted to the PDB format using the Open Babel GUI. For molecular docking, retinol dehydrogenase was used as a receptor, and its molecular ligand was retinal. The docking of the flexible ligand to the rigid receptor was performed using Discovery Studio 2016. The docking results and structural changes were analyzed using PyMOL software 2.2.0 from Schrodinger (New York, NY, USA). To select the target residues for saturation mutations in the catalytic domain, the allosteric sites and pathways were predicted using the online analysis tool Ohm (https://dokhlab.med.psu.edu/ohm (accessed on 7 October 2022).

### 2.5. Culture and Fermentation Conditions

For shake-flask fermentation, we dipped an inoculation ring in *S. cerevisiae* suspension stored in a glycerol tube, drew a line on the YPD solid plate, and cultured it in a 30 °C incubator for 24–48 h. Using an inoculation ring, a single *S. cerevisiae* colony was transferred into 3 mL of YPD liquid medium and cultured at 30 °C with shaking at 220 rpm for 24 h to obtain the seed liquid. According to the amount of inoculation (2–5%), 1 mL of the seed liquid was used to switch to the 30 mL fermentation medium and continuously cultured at 30 °C and 220 rpm for 120 h. Moreover, as chemically active retinol can easily decompose upon light exposure [18], the light source was shielded during fermentation.

### 2.6. Extraction and Detection Methods

The 120 h fermented *S. cerevisiae* suspension was centrifuged, and an upper extractant was taken to determine extracellular level products. The yeast cells were disrupted using FastPrep from MP Biomedicals (Irvine, CA, USA), and the intracellular product was extracted with the same volume of ethyl acetate as the fermentation broth.

The products were purified using the Agilent ZORBAX Eclipse XDB-C18 separation column (5 µm, 250 × 4.6 mm). Retinol and retinal were detected in a mobile phase consisting of methanol and acetonitrile in a ratio of 95:5 at the detection temperature of 25 °C with the detection wavelength of 352 nm. The mobile phase consisted of methanol, acetonitrile, and isopropanol in a ratio of 3:5:2, and the detection temperature was 40 °C [19]. The detection wavelength used for β-carotene was 450 nm. [20]. Squalene was detected at 210 nm using mobile phase methanol at 40 °C [21]. An ultraviolet/visible spectrophotometer was used to determine the absorbance value (OD_600_) of the fermentation broth and detect the growth of *S. cerevisiae*.

### 2.7. Statistical Analysis

All experiments were independently carried out at least three times. Origin was used for statistical analysis. The statistical evaluations used one-way ANOVA, followed by Dunnett tests. Statistical significance is indicated as follows: * *p* < 0.05, ** *p* < 0.01.

## 3. Results and Discussions

### 3.1. Construction of the De Novo Retinol Synthesis Pathway in S. cerevisiae

**Figure 1:**
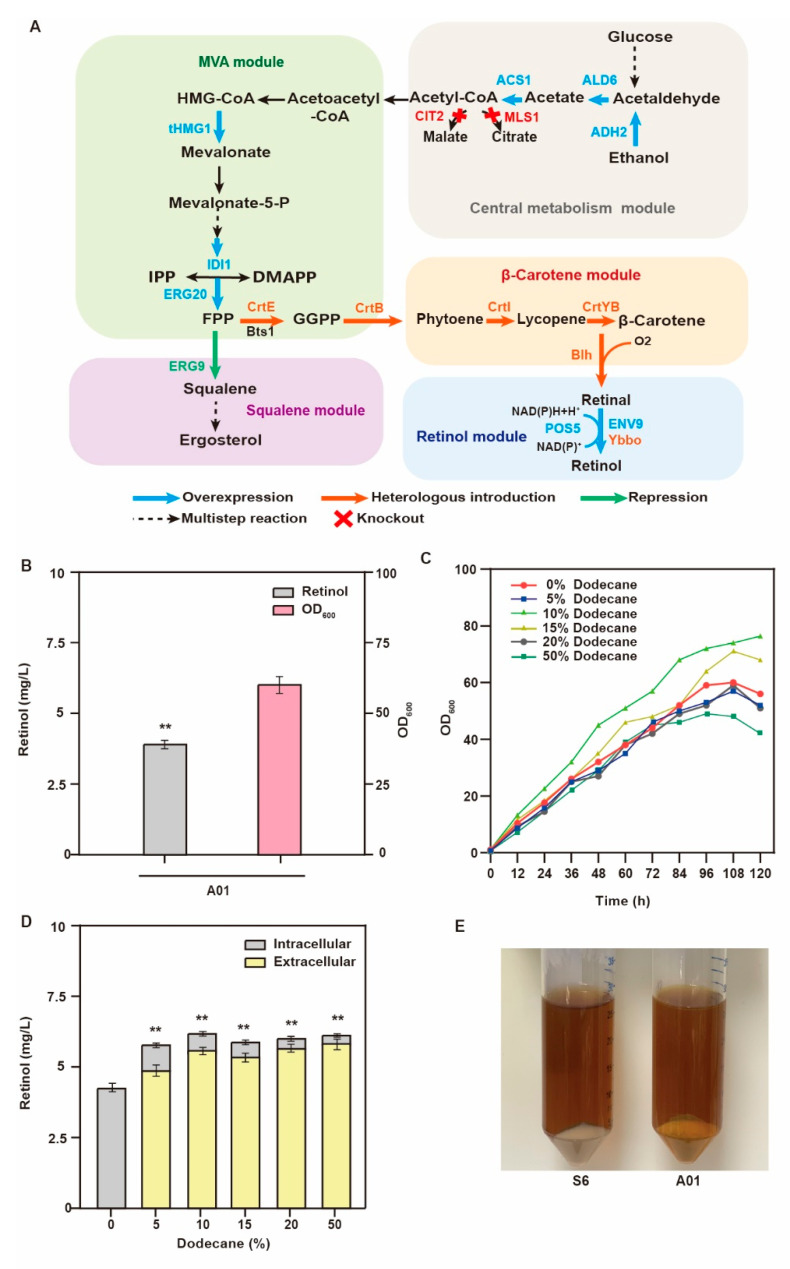
Overview of the retinol biosynthesis pathway and engineering strategy. (**A**) Retinol synthesis by *S. cerevisiae* includes endogenous mevalonate, central carbon metabolism, squalene synthesis, exogenous β-carotene synthesis, retinol modules, and overexpression of endogenous aldehyde dehydrogenase (ALD6), acetyl-CoA ligase (ACS1), ethanol dehydrogenase (ADH2), retinol dehydrogenase (ENV9), and NADH kinase (POS5), as well as exogenous GGPP synthase (CrtE) from *Taxus × media*, phytoene synthase (CrtB) from *Pantoea agglomerans*, phytoene desaturase (CrtI) from *Blakeslea trispora*, lycopene cyclase (CrtYB) from *Xanthophyllomyces dendrorhous*, β-carotene dioxygenase (Blh) from *marine bacterium 66A03*, retinol dehydrogenase Ybbo, knockout citrate synthase (CIT2), and malate synthase (MLS1). (**B**) Retinol production and OD_600_ of strain A01 fermented for 120 h. (**C**) The growth trend of strain A01 with different proportions of dodecane. (**D**) The retinol titer from strain A01 with different dodecane concentrations (** *p* < 0.01 according to one-way ANOVA, followed by Dunnett tests). (**E**) The color changes of strain S6 and A01 after 120 h of fermentation.

Retinol is a fat-soluble vitamin, and excessive accumulation has a toxic effect on cells. The use of constitutive promoters will increase the burden of cell metabolism, while inducible promoters can release the coupling of cell growth and production. Before the inducer is added, the cells only grow, and the genes related to production are not expressed. When the cell concentration reaches a certain level, the inducer is added to activate the expression of related enzymes [22]. We used the inducible promoter P*GAL7* to heterologously express five essential genes of the retinol synthesis pathway in the original strain S6, a precursor FPP accumulation strain preserved in our library (Figure 1A). The de novo retinol synthesis pathway was successfully constructed in *S. cerevisiae* (strain A01), and the total titer of retinol in the cell reached 3.6 mg/L. The galactose-induced promoter P*GAL7* was inhibited by glucose and activated by galactose. However, compared with glucose, the addition of galactose as a carbon source was more difficult to utilize and expensive. Thus, we removed galactose dependence by knocking out the transcription inhibitor GAL80 [23]. As a result, the transcription of GAL was initiated by glucose consumption from the medium (Figure 1B). Hu et al. and Sun et al. found that adding dodecane to the culture medium as an extractant could improve retinol production [9,12]. Therefore, we added different concentrations of dodecane (5%, 10%, 15%, 20%, and 50% [*v*/*v*]) to detect its effects on cell growth and retinol production. Our experimental results revealed that the addition of 10% dodecane was the most beneficial for cell growth (Figure 1C) [12]. There was no linear relationship between dodecane concentration and retinol production, and most of the retinol existed outside the cell (Figure 1D). Upon addition of 10% (*v*/*v*) dodecane, the retinol titer increased to 6.3 mg/L, and the color of the strain also became more yellow (Figure 1E). Therefore, the subsequent experiments were performed using two-phase fermentation with dodecane.

### 3.2. Module Engineering of the Retinol Metabolism Network

To systematically optimize the complex retinol metabolism network, we divided this network into the following five modules: mevalonate synthesis, squalene synthesis, central carbon metabolism, exogenous β-carotene synthesis, and retinol synthesis.

**Figure 2:**
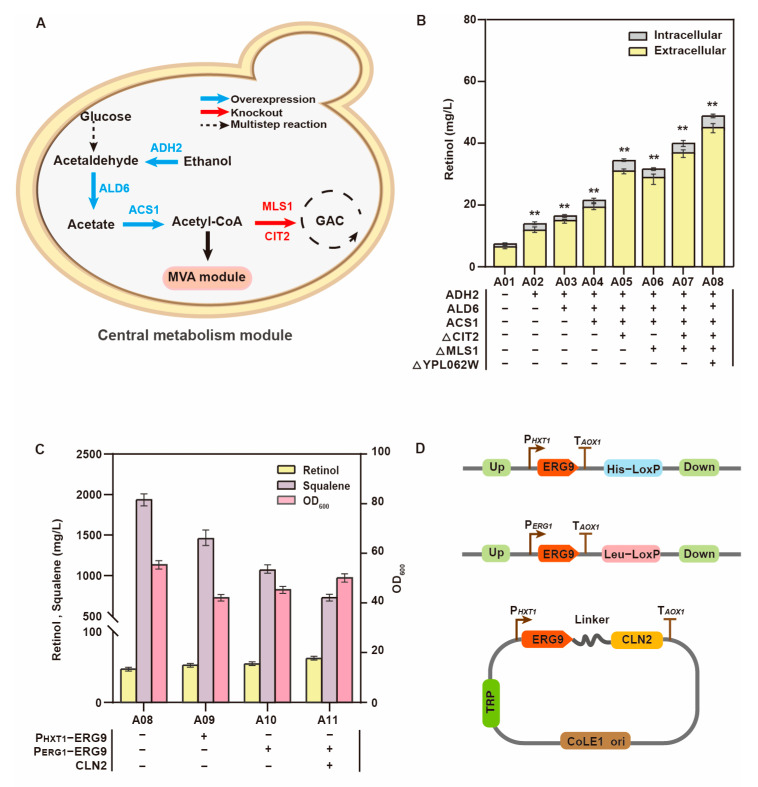
Strengthening the central carbon metabolism module and weakening the competitive branch of squalene synthesis. (The number of plus signs represents the number of gene copies added, and the minus sign represents that no genetic manipulation) (**A**) Central carbon metabolism module pathway. (**B**) Overexpression of the key genes involved in central carbon metabolism affects retinol production (** *p* < 0.01). (**C**) Effect of replacing the natural promoter of ERG9 with HXT1 promoter or ERG1 promoter and adding degradation label CLN2 at the carbon end of ERG9 to reduce the expression of ERG9 on squalene and retinol production and OD_600_. (**D**) Strategies for weakening squalene synthesis (“up” refers to the upstream homologous arm; “down” refers to the downstream homologous arm).

First, we increased the concentration of the precursor acetyl-CoA in the central carbon metabolism module (Figure 2A). Strain A03, which overexpresses alcohol dehydrogenase (ADH2) and acetaldehyde dehydrogenase (ALD6), displayed an improved utilization rate of ethanol. This enhanced acetyl-CoA supplementation, which increased the retinol titer to 17.8 mg/L. To further improve the metabolic pool of acetyl-CoA, we overexpressed acetyl-CoA synthetase ACS1 in strain A04, and the retinol titer reached 22.6 mg/L. In addition, we reduced acetyl-CoA consumption by knocking out *MLS1* and *CIT2*, the key genes involved in the acetyl-CoA reaction in the glyoxylate cycle in strain A07, which was more efficient than strains with individual gene knockouts (strains A05 and A06). The retinol titer in strain A07 reached 38.2 mg/L. Furthermore, knocking out *YPL062W*, an endogenous nonessential gene in *S. cerevisiae*, enhanced the ability of cells to use acetic acid, which weakened the inhibitory effect of acetic acid on cell growth [24]. The resulting strain A08 could produce 45.8 mg/L retinol and 1898.5 mg/L squalene (Figure 2B,C).

However, excess accumulation of the byproduct squalene would hinder retinol synthesis. Therefore, we weakened the metabolic flux of the squalene synthesis module and enhanced the metabolic flux of the β-carotene synthesis module. FPP is converted into squalene by squalene synthase ERG9 [25]; hence, its gene *ERG9* is necessary for normal cell growth. As *ERG9* cannot be directly knocked out, we used the glucose-inducible promoter P*HXT1* to dynamically regulate *ERG9* expression (Figure 2D). When glucose is present in the culture medium, *S. cerevisiae* preferentially uses glucose as the carbon source, which activates P*HXT1*, resulting in the expression of squalene synthase (ERG9). However, when the glucose supply is exhausted, P*HXT1* is inhibited and *ERG9* is not expressed. After replacing the promoter, the intracellular squalene titer of strain A09 decreased from 1898.5 mg/L to 1480.8 mg/L, while its retinol titer reached 54.9 mg/L. The decrease in squalene slightly reduced the biomass of *S. cerevisiae* (Figure 2C). We also replaced the P*ERG9* promoter with P*ERG1* and compared the effect on squalene synthesis. Squalene oxygenase encoded by *ERG1* is the rate-limiting enzyme for the synthesis of ergosterol. The ergosterol reaction promoter P*ERG1* can dynamically control *ERG9* expression (strain A10) to maintain appropriate sterol levels. This helps achieve the highest cell growth in the changing environment, facilitating retinol synthesis. Strain A10 synthesized 1263.7 mg/L squalene and 59.1 mg/L retinol. However, there was significant squalene accumulation in strain A10. Therefore, we fused a protein degradation tag CLN2 [26] into the carboxyl end of ERG9 to decrease its expression (strain A11). The squalene titer in strain A11 decreased from 1263.7 mg/L to 783.9 mg/L, while the retinol titer reached 64.6 mg/L (Figure 2D).

**Figure 3:**
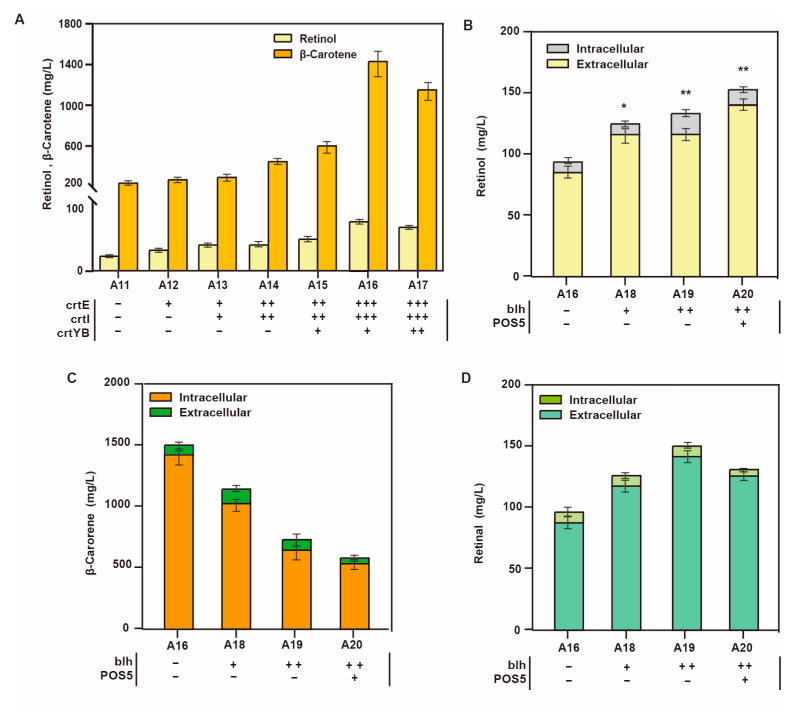
Changes in external β-carotene and retinol synthesis modules. (The number of plus signs represents the number of gene copies added, and the minus sign represents that no genetic manipulation) (**A**) The effect of increasing the copy numbers of the key genes *crtE, crtI,* and *crtYB* in the β-carotene biosynthesis pathway on intracellular β-carotene and extracellular retinol production. Effect of overexpression of the retinol biosynthesis pathway genes *blh* and *POS5* on (**B**) retinol titer (* *p* < 0.05, ** *p* < 0.01 according to one-way ANOVA, followed by Dunnett tests), (**C**) precursor β-carotene production, and (**D**) retinal production (the number of plus signs represents increased gene copies).

The increase in retinol is far less than the decrease in squalene production, possibly due to the rate-limiting steps in the exogenous β-carotene synthesis module. Next, we added multiple copies of the genes *crtE*, *crtI,* and *crtYB* from the β-carotene synthesis module to eliminate these rate-limiting steps. Figure 3A shows the effect of different gene copy numbers on β-carotene and retinol production. The highest β-carotene and retinol production was obtained by increasing three copies of *crtE* and *crtI* and one copy of *crtYB*. The resulting strain A16 produced 1508.7 mg/L β-carotene and 83.6 mg/L retinol (Figure 3B); β-carotenes were mostly intracellular.

Although strain A16 could efficiently produce β-carotene, the retinol titer was still not high, which might have been mitigated by strengthening the metabolic flux of the retinol synthetic module. One molecule of β-carotene produces two retinal molecules under the action of β-carotene-15,15’-dioxygenase (Blh). We obtained strain A19 by increasing two copies of *blh*, which resulted in a significant decline in the titers of β-carotene from 1508.7 mg/L to 728.8 mg/L (Figure 3C). The titer of retinol increased from 83.6 mg/L to 131.4 mg/L.

In the retinol synthesis module, catalysis by retinol dehydrogenase requires NADPH to provide reducing power. NADPH is generated when NAD^+^/NADH receives a phosphate group from ATP via mitochondrial kinases. As the activity of NADH kinase POS5 is crucial for NADPH synthesis [27], we created strain A20 that overexpressed *POS5* and produced 153.6 mg/L retinol.

However, there is still a significant gap between the decrease in β-carotene and the increase in retinol production. Moreover, 127.1 mg/L of extracellular level retinal was detected (Figure 3D), indicating the outflow of the precursor retinal from the cells in large quantities, which hampered retinol synthesis. To reduce the accumulation of extracellular retinal, we adopted the following approaches: inhibited the export of intracellular retinal, screened and strengthened the expression of retinol dehydrogenase, and improved the oxidation resistance of retinol.

### 3.3. Knockout of Retinal Transporter

**Figure 4:**
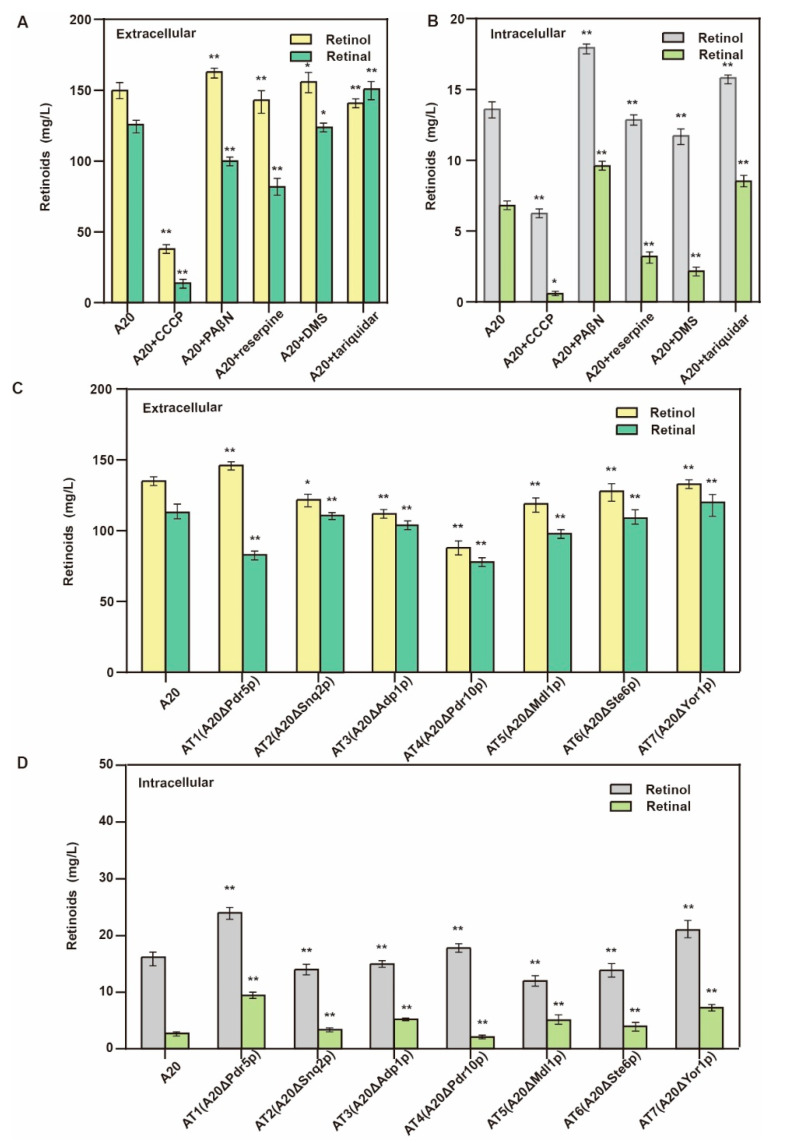
Knockout of ABC transporter inhibits retinol efflux and increases retinol production. (**A**) Addition of ABC transporter inhibitor to verify the effect of ABC transporter on retinoid production at extracellular level. (**B**) Addition of ABC transporter inhibitor to verify the effect of ABC transporter on intracellular retinoid production. (**C**) Effect of knockout of ABC transporter on retinoid production at extracellular level. (**D**) Effect of knockout of ABC transporter on intracellular retinoid production (* *p* < 0.05, ** *p* < 0.01 according to one-way ANOVA, followed by Dunnett tests).

Retinoids are heterogeneous hydrophobic compounds in *S. cerevisiae*. Accumulated retinoids might be incorporated into the membrane, damaging the membrane structure and protein activity, which can be detrimental to cell growth. *S. cerevisiae* has a detoxification mechanism to remove toxic substances from the cell using an efflux pump. The significant outflow of retinal from the cell prevents retinol accumulation. Studies have shown that ABC transporters are essential for the transport of terpenoids in *S. cerevisiae*. The ABC transporter is a type of transmembrane protein that utilizes the energy released by ATP hydrolysis to transport substrates [28]. We used five common ABC transporter inhibitors: carbonyl cyanide chlorophenylhydrazone (CCCP), PAβN, reserpine, dexamethasone (DMS), and tariquidar [29] to preliminarily verify whether the retinal is excreted to the outside through the ABC transporter. The ABC transporter inhibitors inhibit substrate transport by blocking efflux pump energy sources or binding sites on competitive or noncompetitive binding transporters [30,31,32]. After the addition of the inhibitor PAβN, the extracellular secretion of the retinal was reduced by 21.9%, and no inhibition of retinol efflux was observed. Intracellular and extracellular level retinol production increased by 26% and 9%, respectively. Results indicated that the ABC transporter might be involved in the transport of retinal. The intracellular accumulation of the precursor retinal had a positive effect on the synthesis of retinol (Figure 4A,B).

Subsequently, we knocked out seven terpenoid exporters (Pdr5p, Snq2p, Adp1p, Pdr10p, Mdl1p, Ste6p, and Yor1p) to obtain the strains AT1, AT2, AT3, AT4, AT5, AT6, and AT7, respectively. The secretion of retinal was reduced after the knockout of Pdr5p and Pdr10p, but the extracellular level of retinol was also reduced after the knockout of Pdr10p, indicating that Pdr10p might have the ability to transport retinol and retinal. In strain AT1, retinol was increased at both the intracellular and the extracellular level, and the total retinol production was 164.6 mg/L (Figure 4C,D). Moreover, intracellular retinal production increased by 170% and reached 8.5 mg/L, whereas the extracellular level of retinol decreased by 26% (Figure 4D), indicating that Pdr5p might participate in retinal transport.

### 3.4. Screening and Directed Evolution of Retinol Dehydrogenase

**Figure 5:**
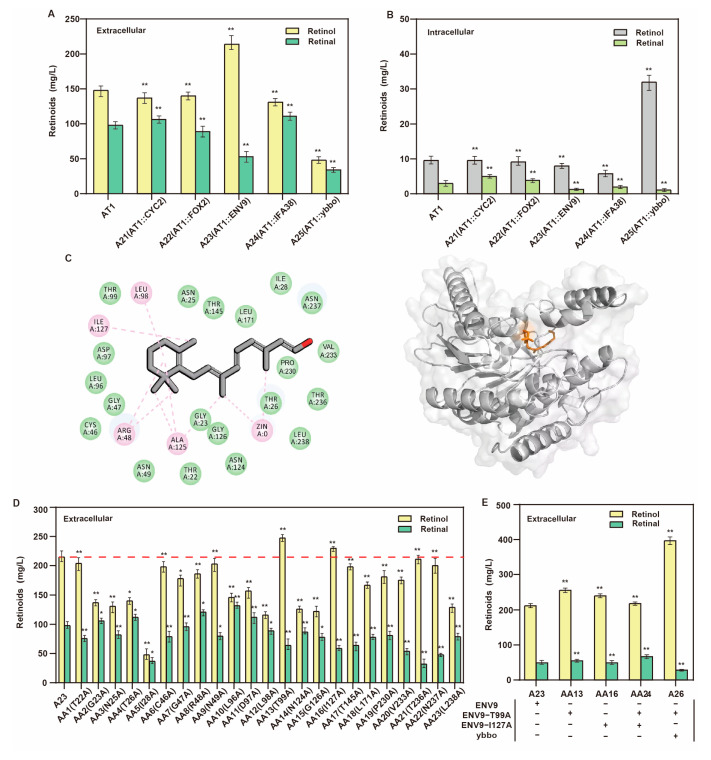
Mining and modification of retinol dehydrogenase to increase retinol production. Effect of different retinol dehydrogenases on (**A**) retinoid production at extracellular level and (**B**) intracellular retinoid production. (**C**) Molecular docking of ENV9 binding to retinal. The left panel represents the key site for molecular docking prediction, the right panel represents the docking of substrate with protein, the gray panel represents protein ENV9, and the orange panel represents substrate retinal. (**D**) Retinoid production at extracellular level change after mutation of key sites on ENV9 (The red dotted line represents the retinol titer of control strain A23). (**E**) Effect of multiple expression of key retinol dehydrogenase on retinoid production at extracellular level (The number of plus signs represents the number of gene copies added, and the minus sign represents that no genetic manipulation; * *p* < 0.05, ** *p* < 0.01 according to one-way ANOVA, followed by Dunnett tests).

As a considerable amount of retinal was still not converted into retinol, the expression of the enzymes related to retinol dehydrogenase activity in *S. cerevisiae* needs to be strengthened to improve the conversion efficiency. By expressing the human *RDH12* gene in *S. cerevisiae*, retinol could be selectively produced, and retinal production could be eliminated [10]. According to the BLASTp function of NCBI, the RDH12 protein sequence in *S. cerevisiae* is homologous to four different dehydrogenase genes, namely, *CYC2*, *FOX2*, *ENV9*, and *IFA38* (31.8%, 30.13%, 30.09%, and 26.56% homology). In addition, *ybbo* from *E. coli* reduces retinal to retinol [33]. Therefore, we overexpressed four endogenous retinol dehydrogenase genes, namely, *CYC2*, *FOX2*, *ENV9*, and *IFA38*, and the exogenous retinol dehydrogenase gene *ybbo* to obtain the recombinant strains A21 to A25, respectively. Overexpression of ENV9 (strain A23) significantly increased the production of retinol at an extracellular level, which reached 214.8 mg/L (Figure 5A). Interestingly, although extracellular level retinol was decreased considerably after *ybbo* overexpression, intracellular retinol was increased from 9.8 mg/L to 32.9 mg/L, and retinal was not detected in the cells (Figure 5B). This result indicated that *ybbo* is involved in retinol accumulation and improves the conversion rate of retinol in cells.

To further improve the retinol titer, we prepared a semi-rational design of the enzyme ENV9. First, the substrate retinal was attached with ENV9 to predict 23 key sites, namely, THR22, GLY23, ASN25, THR26, ILE28, CYS46, GLY47, ARG48, ASN49, LEU96, ASP97, LEU98, THR99, ASN124, GLY126, ILE127, THR145, LEU171, PRO230, VAL233, THR236, ASN237, and LEU238 (Figure 5C). The abovementioned sites were mutated into alanine and expressed in strain AT1 to produce strains AA1 to AA23, respectively. We found that strain AA13 (T99A) produced 254 mg/L retinol and 55.2 mg/L retinal, and strain AA16 (I127A) produced 238 mg/L retinol and 48.7 mg/L retinal (Figure 5D). However, the effect of iterative mutation of these two mutation sites was not as good as that of a single mutation. Lastly, the mutants ENV9 and Ybbo were simultaneously expressed to obtain strain A26, which produced 387.4 mg/L retinol (Figure 5E).

### 3.5. Expansion of the Storage Space for Lipid Droplets

As retinal and retinol are mostly stored in lipid droplets, their accumulation might cause a metabolic burden on cells [34]. Therefore, expanding the lipid droplet space is crucial for efficient retinol synthesis. Lipid droplets in eukaryotes are composed of a hydrophobic neutral lipid core and a phospholipid monolayer. The most important neutral lipids in the nucleus are triacylglycerol (TAG) and sterol ester (SE). The phospholipids in the lipid drop membrane are mainly phosphatidylcholine (PC), phosphatidylinositol (PI), and phosphatidylethanolamine (PE), whose side-chains are rich in unsaturated fatty acids.

**Figure 6:**
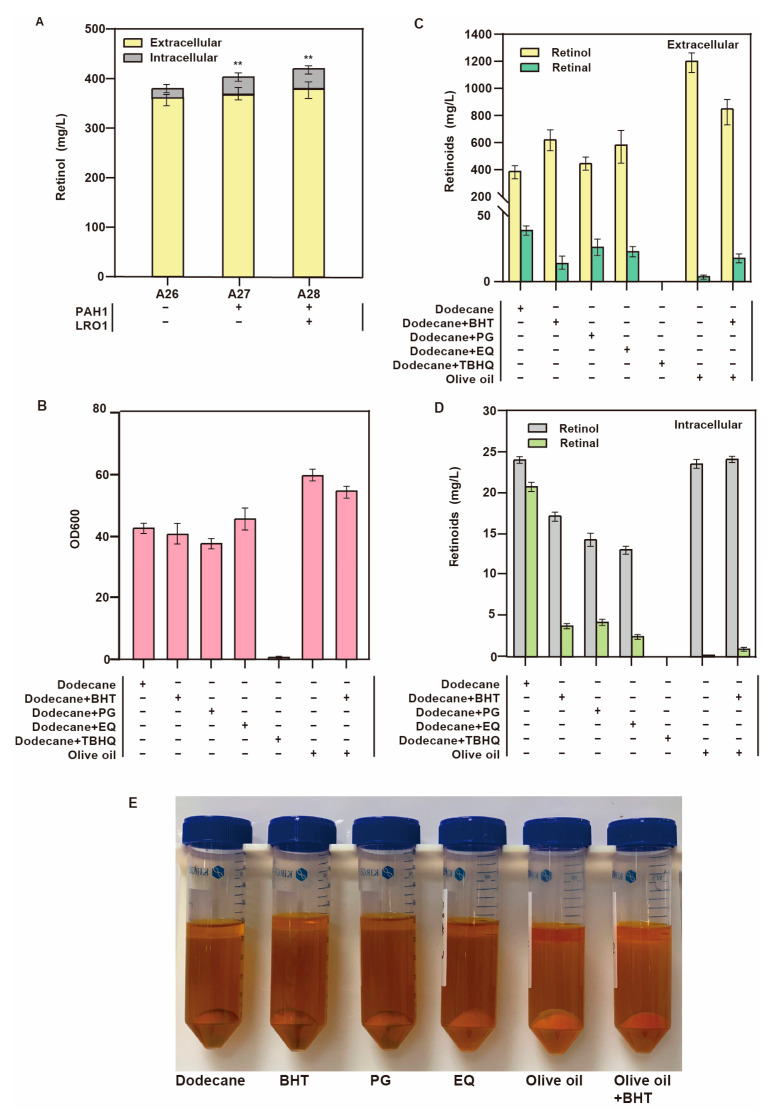
Increasing intracellular storage space and enhancing antioxidant activity to promote retinol production. (The number of plus signs represents the number of gene copies added, and the minus sign represents that no genetic manipulation) (**A**) The effect of overexpressing genes related to lipid droplet synthesis on retinol production at intracellular and extracellular levels (** *p* < 0.01 according to one-way ANOVA, followed by Dunnett tests). (**B**) Effect of adding different antioxidants on the growth of strain A28. (**C**) Effect of adding different antioxidants on retinoid production at extracellular level by strain A28. (**D**) Effect of adding different antioxidants on intracellular retinoid production by strain A28. (**E**) Effect of adding different antioxidants on color change of strain A28.

We overexpressed phosphatidate phosphatase PAH1 involved in TAG synthesis, and the retinol production of strain A27 increased from 387.4 mg/L to 412 mg/L. Overexpression of diacylglycerol acyltransferase LRO1, which is involved in SE synthesis, increased the intracellular retinol production from 19.4 mg/L to 40.2 mg/L, with a total retinol titer of 425.7 mg/L in strain A28 (Figure 6A).

### 3.6. Improvement of Oxidation Resistance

In addition to using metabolic engineering to improve the performance of strains, fermentation conditions can be optimized to enhance retinol production. Retinol is an unsaturated monohydric alcohol, and its side-chain contains four conjugated double bonds, making it relatively chemically active. The hydroxymethyl at its end can be easily oxidized to an aldehyde group, which rapidly decomposes when exposed to light. The addition of dodecane as an extractant promotes extracellular level retinol accumulation [9], but retinol will still be oxidized to retinal in dodecane. As the presence of dodecane might inhibit the conversion of retinal to retinol, we chose two strategies to improve this conversion. The first was adding antioxidants into the extractant, and the second was using a more suitable extractant.

As an additive, antioxidants can help capture and neutralize free radicals, thereby removing free radicals and delaying the oxidation and decomposition of substances [35]. On the basis of their mechanism of action, antioxidants can be divided into chelating agents, free-radical scavengers, and reduction protectors. To reduce retinol oxidation, we selected four different free-radical scavengers, namely, butylated hydroxytoluene (BHT), propyl gallate (PG), ethoxy quinoline (EQ), and tert-butyl hydroquinone (TBHQ). BHT is a highly stable colorless crystal whose antioxidant effect is not affected by heat and can effectively delay the oxidation of fat-soluble substances. EQ is a yellow viscous liquid, almost insoluble in water, has a long-lasting effect, is easily miscible, has strong dispersion, and is an excellent antioxidant to protect vitamins [36]. We added 1% (*v*/*v*) antioxidants to the extractant to verify their effect on the retinol titer using the dodecane medium without antioxidants as the control group. TBHQ addition affected the growth of the strain, and retinol production was not detected. The addition of the other three antioxidants had no significant effect on cell growth (Figure 6B), and the retinol titer was increased. BHT and EQ elicited significantly higher retinol production than other antioxidants. The addition of BHT and EQ to strain A28 resulted in a retinol titer of 621.3 mg/L and 582.6 mg/L, respectively (Figure 6C). In both cases, the retinal titer of strain A28 was less than 20 mg/L. These results revealed that the addition of antioxidants delayed retinol oxidation and increased its titer and the rate of conversion of retinal to retinol, which undoubtedly enhanced the retinol titer.

Herein, our aim was not only to increase retinol production but also to solve the food safety problems associated with toxic extractants. We used olive oil instead of dodecane as the extractant. Olive oil, which is rich in oleic acid, contains over 98% triglycerides and 2% bioactive substances [37], making it an excellent antioxidant. Olive oil could form anaerobic conditions during the fermentation of *S. cerevisiae*, thereby improving retinol accumulation. Here, we also found that olive oil extraction was beneficial to the growth and production of strains. After strain A28 was fermented for 120 h, the retinol titer reached 1.2 g/L (Figure 6C). No extracellular retinal was detected, and only 19.3 mg/L of intracellular retinal was observed. The conversion rate of retinal to retinol was over 98% (Figure 6D).

In summary, the use of dodecane with antioxidant or olive oil alone for extraction has unique advantages. We combined olive oil and dodecane at a ratio of 1:1 to extract retinol, and the retinol yield reached 849 mg/L. We also observed apparent color differences using the two extraction methods. The color of the olive oil extract supernatant was darker, indicating that it contained more retinol (Figure 6E). The results showed that the effect of extracting olive oil alone was the best, better than that of olive oil and dodecane compound extraction with BHT, while the effect of adding BHT in dodecane was the worst.

## 4. Conclusions

In this study, the de novo synthesis pathway of retinol was constructed in *S. cerevisiae.* A highly efficient retinol-producing strain was successfully constructed using systematic metabolic engineering, transporter engineering, semi-rational transformation of enzymes, and optimization of fermentation conditions. The final retinol titer reached 1.2 g/L after fermentation in a 250 mL shake flask, indicating efficient retinol production. We intend to further improve the stability and establish long-term effective preservation methods of retinol in our future studies.

## Figures and Tables

**Table 1:** *Saccharomyces cerevisiae* strains used in this study.

Strains	Characteristics	Reference
*Escherichia coli* JM109	*recA1*, *endA1*, *thi*, *gyrA96*, *supE44, hsdR17*△ (*lac-proAB*)*/F* [*traD36*, *proAB*+*, laclq, lacZ*△*M15*]	Lab stock
*S. cerevisiae* CEN.PK2-1C	*MATa*, *his3D1*, *leu2-3_112*, *ura3-52, trp1-289*, *MAL2-8c*, *SUC2*	Lab stock
S6	*S. cerevisiae* CEN.PK2-1C derivate, ∆*911b*:: P*_TEF1_*-*IDI*, ∆P*_INO2_*:: P*_PGK_*-P*_TEF1_*-*ERG20-P_GPD_-tHMG1*, ∆*ROX1*:: P*_GPD_*-*tHMG1*	Jin et al. [17]
A01	S6 derivate, ∆*308a*:: P*_GAL7_*-*crtE*, ∆*607c*:: P*_GAL7_*-*crtB*, ∆*GAL80*:: P*_GAL7_*-*crtI*, ∆*1309a*:: P*_GAL7_*-*crtYB*, ∆*1014a*:: P*_GAL7_*-*blh*	This study
A02	A01 derivate, ∆*1021b*:: P*_TEF1_*-*ADH2*	This study
A03	A02 derivate, ∆*HIS3b*:: P*_TEF1_*-ALD6	This study
A04	A03 derivate, ∆*805a*:: P*_TEF1_*-*ACS1*	This study
A05	A04 derivate, ∆*CIT2*	This study
A06	A05 derivate, ∆*MLS1*	This study
A07	A06 derivate, ∆*CIT2* ∆*MLS1*	This study
A08	A07 derivate, ∆*YPL062W*	This study
A09	A08 derivate, P*_ERG9_*:: P*_HXT1_*	This study
A10	A08 derivate, P*_ERG9_*:: P*_ERG1_*	This study
A11	A08 derivate, P*_ERG9_*:: P*_ERG1_*-*ERG9*-*CLN2*	This study
A12	A11 derivate, ∆*1414a*:: P*_GAL7_*-*crtE*	This study
A13	A11 derivate, ∆*1414a*:: *crtE*-P*_GAL1,10_*-*crtI*	This study
A14	A13 derivate, ∆*720a*:: *crtE*-P*_GAL1,10_*-*crtI*	This study
A15	A14 derivate, ∆*1114a*:: P*_GAL7_*-*crtYB*	This study
A16	A15 derivate, ∆*416d*:: *crtE*-P*_GAL1,10_*-*crtI*	This study
A17	A16 derivate, ∆*106a*:: P*_GAL7_*-*crtYB*	This study
A18	A16 derivate, ∆*1206a*:: P*_GAL7_*-*blh*	This study
A19	A16 derivate, ∆*1206a*:: *blh*-P*_GAL1,10_*-*blh*	This study
A20	A19 derivate, ∆*CAN1y*:: P*_GAL7_*-*POS5*	This study
AT1	A20 derivate, ∆*Pdr5*	This study
AT2	A20 derivate, ∆*Snq2*	This study
AT3	A20 derivate, ∆*Adp1*	This study
AT4	A20 derivate, ∆*Pdr10*	This study
AT5	A20 derivate, ∆*Mdl1*	This study
AT6	A20 derivate, ∆*Ste6*	This study
AT7	A20 derivate, ∆*Yor1*	This study
A21	AT1 derivate, ∆*1622b*:: P*_GAL7_*-*CYC2*	This study
A22	AT1 derivate, ∆*1622b*:: P*_GAL7_*-*FOX2*	This study
A23	AT1 derivate, ∆*1622b*:: P*_GAL7_*-*ENV9*	This study
A24	AT1 derivate, ∆1622b:: P*_GAL7_*-*IFA38*	This study
A25	AT1 derivate, ∆*1622b*:: P*_GAL7_*-*ybbo*	This study
A26	AT1 derivate, ∆*1622b*:: P*_GAL7_*-*ENV9* (T99A), ∆*208a*:: P*_GAL7_*-*ybbo*	This study
A27	A26 derivate, :: P*_TEF1_*-*PAH1*	This study
A28	A27 derivate, :: P*_TEF1_*-*LRO1*	This study

## Data Availability

Not applicable.

## Supplementary Materials

The following supporting information can be downloaded at https://www.mdpi.com/article/10.3390/jof9050512/s1: Table S1. Plasmids used in this study.
