# Supplementary material for: Metabolic Engineering of Saccharomyces cerevisiae for Efficient Retinol Synthesis
Source: J Fungi (Basel). 2023 Apr 26;9(5):512. doi: 10.3390/jof9050512 (PMC10219262; doi:10.3390/jof9050512)
Supplement: Supplementary file 1 [file jof-09-00512-s001.zip › jof-2295632-supplementary.pdf]

Supplementary Materials

Table S1. Plasmids used in this study

Plasmids	Characteristics	Reference
PY13-crtE	PY13 derivate, PGAL7-crtE	This study
PY14-crtI	PY14 derivate, PGAL7-crtI	This study
PY15-crtB	PY15 derivate, PGAL7-crtB	This study
PY13-crtYB	PY13 derivate, PGAL7-crtYB	This study
PY14-blh	PY14 derivate, PGAL7-blh	This study
PY15-blh-blh	PY15 derivate, blh-PGAL1,10-blh	This study
PY13-crtE-crtI	PY13 derivate, crtI-PGAL1,10-crtE	This study
PY13-ERG9	PY13 derivate, PHXT1-ERG9	This study
PY15-ERG9	PY15 derivate, PERG1-ERG9	This study
PY14-ERG9	PY14 derivate, PHXT1-ERG9-CLN2	This study
PY26-POS5	PY26 derivate, PGAL7-POS5	This study
PY13-CYC2	PY13 derivate, PGAL7-CYC2	This study
PY13-FOX2	PY13 derivate, PGAL7-FOX2	This study
PY13-ENV9	PY13 derivate, PGAL7-ENV9	This study
PY13-IFA38	PY13 derivate, PGAL7-IFA38	This study
PY13-ybbo	PY13 derivate, PGAL7-ybbo	This study
PY15-PAH1	PY15 derivate, PTEF1-PAH1	This study
PY15-LRO1	PY15 derivate, PTEF1-LRO1	This study
